# Apolipoprotein E gene polymorphism influences aggressive behavior in prostate cancer cells by deregulating cholesterol homeostasis

**DOI:** 10.3892/ijo.2013.2057

**Published:** 2013-08-07

**Authors:** GODWIN O. IFERE, RENEE DESMOND, WENDY DEMARK-WAHNEFRIED, TIM R. NAGY

**Affiliations:** 1 Department of Nutrition Sciences, University of Alabama at Birmingham;; 2 Division of Preventive Medicine, UAB, Birmingham, AL 35294, USA

**Keywords:** prostate cancer, apolipoprotein E, alleles, cholesterol, aggressive, caveolin-1, polymorphism, genotype, phenotype

## Abstract

High circulating cholesterol and its deregulated homeostasis may facilitate prostate cancer progression. Genetic polymorphism in Apolipoprotein (Apo) E, a key cholesterol regulatory protein may effect changes in systemic cholesterol levels. In this investigation, we determined whether variants of the Apo E gene can trigger defective intracellular cholesterol efflux, which could promote aggressive prostate cancer. ApoE genotypes of weakly (non-aggressive), moderate and highly tumorigenic (aggressive) prostate cancer cell lines were characterized, and we explored whether the ApoE variants were associated with tumor aggressiveness generated by intra cellular cholesterol imbalance, using the expression of caveolin-1 (cav-1), a pro-malignancy surrogate of cholesterol overload. Restriction isotyping of ApoE isoforms revealed that the non-aggressive cell lines carried ApoE ε3/ε3 or ε3/ε4 alleles, while the aggressive cell lines carried the Apoε2/ε4 alleles. Our data suggest a contrast between the non-aggressive and the aggressive prostate cancer cell lines in the pattern of cholesterol efflux and cav-1 expression. Our exploratory results suggest a relationship between prostate aggressiveness, ApoE isoforms and cholesterol imbalance. Further investigation of this relationship may elucidate the molecular basis for considering cholesterol as a risk factor of aggressive prostate tumors, and underscore the potential of the dysfunctional ApoE2/E4 isoform as a biomarker of aggressive disease.

## Introduction

African-American men have a two-fold risk of mortality from prostate cancer, and currently one of the most unsettled debates is whether this high risk is driven by behavior, biology or both ( 1 , 2 ) . Pathological features, such as larger prostate sizes, greater tumor volumes and tumor grades of African Americans, in contrast to Caucasians strongly implicate biological differences for this disparity ( 3 – 5 ) . In contrast, the disparity in incidence of the disease between second generation Asian migrants and their prostate cancer-free pedigrees strongly implicate environmental or behavioral/lifestyle variables as risk factors in the etiology or prognosis of the disease ( 6 ) . Adoption of a Western lifestyle characterized by less physical activity, increased intake of red meat and dairy products high in saturated fats and cholesterol has been associated with increased risk and poor prognosis of the disease ( 6 – 8 ) . Regardless, epidemiological and population studies reveal that lifestyle or environmental factors alone seldom account for the disparity in cancer risk among different populations ( 9 ) . Current evidence rather suggests that, the interaction of specific genetic and lifestyle factors predispose populations to most cancers ( 10 – 12 ) .

The suggestion that some diets increase the burden of prostate cancer has encouraged the increased scrutiny of cholesterol-rich, Western-style diets as risk factors for the disease. Overwhelming evidence suggests that all direct human ancestors were largely herbivorous, and that the shift to meat or cholesterol-rich diets in our immediate human ancestors selected for genes that modulate the extra cholesterol burden ( 13 ) . One meat-adaptive gene that critically mediates cholesterol and lipid uptake by cells throughout the body is apolipoprotein (Apo) E ( 13 , 14 ) . ApoE displays genetic polymorphism with three common alleles namely, ε2, ε3, and ε4 in a single gene-locus in chromosome 19, giving rise to 3 homo-zygous (apoε2/ε2, apoε3/ε3, apoε4/ε4) and 3 heterozygous genotypes (Apoε2/ε3, Apoε2/ε4, Apoε3/ε4) ( 15 ) . Variants of this gene account for more genetic differences in cholesterol metabolism than any other gene ( 16 , 17 ) . Apoε3 genotype is reportedly selected for its positive effects in cholesterol control and for reducing the risk of various diseases ( 13 ) . Yet again for cholesterol control, the ε2 and ε4 alleles are regarded as dysfunctional forms of the ε3 alleles ( 18 ) , and are considered to enhance the susceptibility to certain cancers ( 19 ) . The ApoE3 gene is the most prevalent in all human populations, and is considered to have evolved from ancestral ApoE 4-like gene ( 13 ) . Based on the different binding affinities of apoE4 and apoE2 to the LDL receptor and the LDL-related protein receptor, rates of postprandial clearance of remnant lipoproteins are reportedly low in subjects with the ApoE2/E2 phenotype, and high in those with the apoE4/E4 phenotype ( 20 ) . Overall, carriers of the ε2 allele have lower serum LDL-cholesterol concentrations relative to carriers of the ε3 and ε4 alleles ( 21 ) .

The ApoE gene also regulates reverse cholesterol transport by transporting the excess cholesterol in peripheral tissues (including the prostate) to the liver for excretion ( 22 ) . Efficient cellular cholesterol efflux depends on ATP-binding cassette transporters (ABCA1 and ABCG1), and uses poor ApoAI and ApoE-containing particles (γ-LpE) from ApoE3/E3 subjects as avid cholesterol acceptors ( 22 ) . In some cells, the transcription of ApoE is induced by exposure to cholesterol, and its release into the extracellular medium is by classical secretory pathway ( 23 ) . There is evidence that secreted ApoE also removes cholesterol in an ABCA1-independent manner ( 24 ) . This pathway requires the intracellular synthesis and transport of ApoE through internal membranes before its secretion ( 24 ) . Regardless of the pathway used for cholesterol efflux, theApoE isoforms differ in their ability to deliver cholesterol to cells, and in their ability to mediate cholesterol efflux ( 25 , 26 ) . This is consistent with the plausible differences in isoform-specific build-up in intracellular cholesterol. Nascent cellular cholesterol first appears in rafts or caveolae domains of plasma membranes, where it forms a complex with caveolin-1 (cav-1), a cholesterol-binding protein ( 27 , 28 ) . The caveolae provide scaffolds for the aggregation of signaling molecules that increase biological activities for cell survival, and cell proliferation ( 29 ) . Also stored cholesterol is vital for mammalian cell growth, as growth requires cholesterol for membrane biogenesis ( 28 ) . Since cav-1 is a growth signaling molecule, it may serve as a surrogate for cell proliferation, which is the major feature of prostate tumor progression. The aim of this study is to explore whether prostate cancer cells with apoE2/E4 phenotypes accumulate higher prostatic tissue cholesterol, and whether cholesterol storage in such cell lines is associated with aggressive cell phenotypes.

## Materials and methods

### Materials

Human prostatic adenocarcinoma cells LNCaP, PC3, DU145 and MDA PCa 2b were obtained from American Type Culture Collection (ATCC, Manassas, VA, USA). Cell culture media, MEM and RPMI-1640 were purchased from Sigma-Aldrich (St. Louis, MO, USA), while HPC1 serum free medium and FNC coating mix were obtained from Athena Enzyme Systems (Baltimore, MD, USA). Sigma-Aldrich supplied all the cell culture supplements, adenosine 3′,5′ cyclic monophosphate (cAMP), acyl-CoA cholesterol acyltransferase (ACAT) inhibitor (Sandoz 58-035), cholic acid, enhanced avian reverse HS RT-PCR kit, and (2-hydroxypropyl)-β-cyclodextrin. BODIPY-cholesterol was purchased from Avanti Polar Lipids, Inc. (Alabaster, AL, USA), while Life Technologies (Grand Island, NY, USA) supplied fetal bovine serum (FBS). Human ApoAI was purchased from BioVision Inc. (Milpitas, CA, USA). Gene ruler ultra-low range DNA ladder and Fast digest *Hha* I restriction enzyme kit was supplied by Fermentas Inc. (Glen Burnie, MD, USA), while Novex TBE running buffer, Hi-density sample buffer and polyacrylamide 8% TBE gels were supplied by Invitrogen (Carlsbad, CA, USA). QIAmp DNA mini kit and RNeasy kit were supplied by Qiagen (Valencia, CA, USA).

### Cell cultures

LNCaP cells were grown in RPMI-1640 medium supplemented with 10% FBS, 1% L-glutamine, 0.5 ml fungizone, 5 ml 100 mM sodium pyruvate, 1% penicillin-streptomycin and buffered with 0.75% HEPES. PC3 and DU145 cells were cultured in MEM with 10% FBS 1% penicillin/streptomycin, 1% glutamine, 1% non-essential amino acids, 0.1% gentamicin and fungizone, and buffered with 0.75% HEPES. MDA PCa 2b cells were grown in HPC1 medium in FNC-precoated 6-well plates. The cells were incubated at 37°C in 5% CO _2_ .

### Amplification of ApoE sequences from genomic DNA

Genomic DNA was extracted as described in QIAMP DNA Mini and blood Mini handbook (Qiagen) from confluent prostate cancer cells. ApoE sequences were amplified from 0.5 *μ* g of genomic DNA using Jump Start AccuTaq DNA polymerase (Sigma-Aldrich), and oligonucleotide primers F4 (5′-ACAGAATTCGCCCCGGCCTGGTACAC-3′) and F6 (5′-TAAGGTTGGCACGGCTGTCCAAGGA-3′) in a Master cycler (Eppendorf) as previously described ( 30 ) . The ApoE sequences were amplified using the enhanced avian reverse transcriptase-PCR kit procedure (Sigma-Aldrich). The reaction was carried out in a reaction mixture volume of 50 *μ* l. Reaction conditions were: 1 cycle of 95°C for 5 min. This was followed by 30 cycles of heating (denaturation) at 95°C for 1 min and annealing at 60°C for 1 min. Extension was at 70°C for 2 min, and final extension was at 70°C for 2 min. To confirm the amplification of the ApoE sequences, the PCR products were analyzed on 1.5% agarose gel and visualized by ethidium bromide staining methods using the Gel Imaging Kodak MI standard Logic. The concentration of PCR products was then measured using NanoDrop 1000 spectrophotometer (Thermo Scientific, Wilmington, DE, USA).

### Restriction isotyping of amplified ApoE sequences with HhaI and gel analysis

After PCR amplification 3 *μ* l of FastDigest *Hha* I enzyme (Fermentas) was added directly to each reaction mixture comprised of 30 *μ* l of PCR amplification product, 51 *μ* l of nuclease free water and 6 *μ* l of 10X FastDigest green buffer. The reaction mixtures were mixed gently and incubated at 37°C in a water bath for 5 min, and then loaded onto 8% non-denaturing pre-cast Novex polyacrylamide TBE gels (1.5-mm thick and 8-cm long) and electrophoresed with 5X Novex TBE running buffer for 20 min. The gel was run in an XCell *Sure* Lock Mini-Cell electrophoresis tank at 200 constant voltage and stopped when the bromophenol tracking dye was approximately two inches from the bottom of the tank to enable visibility of the 20- and 25-bp bands. After electrophoresis, the gel was treated with ethidium bromide (6 *μ* l of 30%) for 10 min, washed three times in distilled water and visualized by UV using the Gel Imaging Kodak MI standard Logic. The sizes of the *Hha* I fragments were estimated by comparison with Ultra low range DNA gene ruler (Fermentas Inc.).

### Cav-1 gene expression analysis by RT-PCR

After seeding all the prostate cancer cell lines to confluence as described above, they were washed with PBS, trypsinized and harvested on ice. RNA was extracted from them according to the procedure described in the RNeasy isolation kit (Qiagen). From each sample, 0.25 *μ* g of total RNA was subjected to first-strand cDNA synthesis using the enhanced avian reverse transcriptase-PCR kit procedure (Sigma-Aldrich). The same amount of each cDNA was used to amplify fragments of cav-1 and GAPDH genes in the presence of Taq DNA polymerase (Sigma-Aldrich). Specific primers for PCR amplification of the two genes were: i) cav-1: sense 5′-CAACAAGGCCATGGCA GACGAGCT-3′ and antisense 5′-CATGGTACAACTGCC CAGATG-3′; (b) GAPDH: sense 5′-AGGTCGGAGTCAACG GATTG-3′ and antisense 5′-GTGATGGCATGGACTGT GGT-3′. Cycling conditions were: one initial denaturation cycle of 95°C for 5 min. This was followed by 38 cycles of denaturation at 95°C for 2 min (for cav-1) or 1 min (for GAPDH), and annealing at 55°C for 2 min (for cav-1) or 57°C for 1 min (for GAPDH). Extension was at 72°C for 2 min and final extension was for 1 cycle at 72°C for 5 min. The amplified products were analyzed on 1.5% agarose gel and visualized by ethidium bromide staining method. The intensity of the bands was quantified with the Gel Imaging Kodak MI standard Logic. All the reactions were normalized with GAPDH mRNA expression.

### Treatment of cells with BODIPY-cholesterol

After growing the cells to confluence in their respective media, they were washed with phosphate buffered saline (PBS), and labeled with a complex mixture of 2 *μ* g/ml ACAT inhibitor, unlabeled cholesterol, BODIPY-cholesterol and 2-hydroxypropyl-β-cyclodextrin (2HβCD), prepared as previously described ( 31 ) . All the cells were incubated in 1 ml of the labeling medium for 1 h, and then washed with their respective HEPES-buffered growth media. The cells were then equilibrated with these growth media containing 0.2% BSA, ACAT inhibitor, and cAMP (0.3 mmol/l) for 18 h. After the equilibration period, the cells were washed with the HEPES-buffered media and triplicates of the experimental groups were incubated for 12 h in the media having cholesterol acceptors (10 *μ* g/ml of ApoA-I), while the control group received no such treatment.

#### Analysis of efflux and intracellular BODIPY-cholesterol

At the end of the incubation period, the efflux media in the control and cholesterol acceptor groups were removed, filtered through a 0.45- *μ* m filter, and the fluorescence intensity measured with Infinite M200 microplate reader (Tecan) at 482-nm excitation and 515-nm emission. To analyze the intracellular concentration of BODIPY-cholesterol, all the cells in the control and cholesterol acceptor groups were solubilized with 1% cholic acid and thoroughly mixed by shaking on a plate shaker for 4 h at room temperature. The fluorescence was again measured as described.

### Microscopy and image analysis

After labeling the cells with BODIPY-cholesterol mixture and subsequent equilibration in the respective growth media, the cells were washed with PBS and treated with cholesterol acceptors as described above. They were then split into two groups; one group was incubated for 12 h in cholesterol acceptors (ApoAI), and the second group had no acceptors. In both groups, the labeled BODIPY-cholesterol was visualized within the living cells using an IX70 inverted microscope (Olympus) equipped with a polychrome IV monochromator (TILL Photonics) with appropriate filters. BODIPY fluorescence intensity in the plasma membrane was then analyzed with Image-Pro Plus software (Cybernetics).

### Statistical analysis

Statistical analyses were performed using Minitab 16 (Minitab Inc., University Park, PA, USA) and SigmaPlot 10.0 (Systat Software Inc., San Jose, CA, USA). Data are presented as the means ± SD, n=3. Differences between the groups were analyzed by a one-way analysis of variance (ANOVA) and by Tukey’s post-hoc test.

## Results

### ApoE genotypes of the prostate cancer cell lines

**Figure 1.**
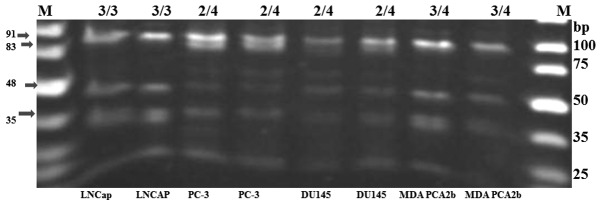
Separation of *Hha* I fragments of amplified ApoE isoforms by polyacrylamide gel electrophoresis. Amplified ApoE genes were from the aggressive and non-aggressive prostate cancer cell lines. The fragment sizes (in bp) are determined from DNA standards marked as M to the left and right of the gel image.

In this study, the unique restriction fragments from the Apoε2 genotypes were detected as the 91- and 83-bp bands, while the fragments for the Apoε3 genotypes aligned with the 91-, 48- and 35-bp bands of the DNA marker. The restriction fragments of the Apoε4 genotypes were identified by their 48- and 35-bp fragments as well as their unique 72-bp fragment. The intensities of the overlapping 35/38-bp bands were lower for all the Apoε2/ε4 restriction fragments because of the absence of the 35 bp fragment in all Apoε2 genotypes. Fig. 1 shows the polyacrylamide gel separation of ApoE isoforms from genomic DNA of prostate cancer cells, after the DNA amplification by PCR, and digestion with the restriction enzyme *Hha* I. The digested fragments revealed the presence of homozygous and heterozygous combinations of Apoε alleles. From the *Hha* I cleavage signature fragments, the LNCaP cell line carried homozygous Apoε3/ε3 alleles, while PC3 and DU145 were both heterozygous for the Apoε2/ε4 alleles. Another heterozygous allele combination (Apoε3/ε4) was found in MDA PCa 2b cell line.

### Relative expression of cav-1 gene in aggressive and non-aggressive prostate cancer cell lines

**Figure 2.**
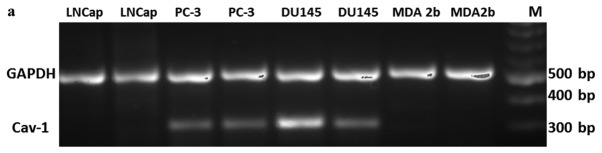

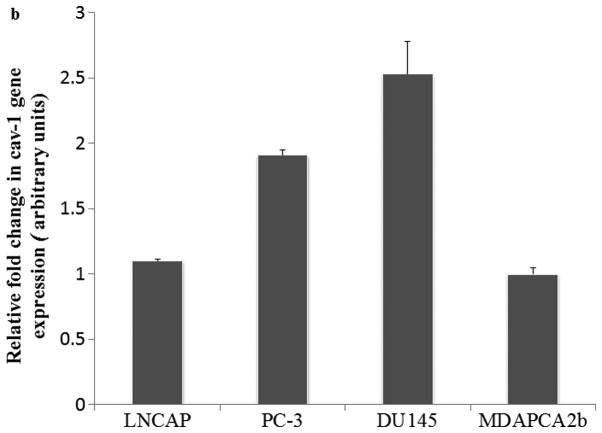
(a) Expression of cav-1 mRNA levels in replicates of different prostate cancer cell lines. The sizes of DNA standard in bp are marked as M. MDA PCA 2b is abbreviated as MDA2b. (b) Semi-quantitative PCR analysis showing the relative fold change in cav-1 gene expression in aggressive and non-aggressive prostate cancer cell lines. Gel electrophoresis bands were quantitated using Kodak Image Station 2000 R and the sum intensities of the bands were normalized to GAPDH.

Endogenous ApoE colocalizes with cav-1 at the plasma membrane to maintain lipid flux ( 32 ) , thus we investigated the relative expression of cav-1 mRNA in the prostate cancer cell lines in order to validate its relationship with variants of ApoE gene and cholesterol balance. Our results showed a relationship between specific ApoE isoforms and the level of expression of cav-1 mRNA in prostate cancer cell lines. According to the agarose gel electrophoresis separation image shown in Fig. 2a , cav-1 mRNA expressed markedly in the aggressive cell lines (PC3 and DU145). By contrast, cav-1 mRNA was undetectable in the non-aggressive cell lines (LNCaP and MDA PCa 2a). Semi-quantitative evaluation of cav-1 expression by RT-PCR showed almost a two-fold increase in cav-1 expression in the aggressive cell lines as compared to the non-aggressive cell lines ( Fig. 2b ). The difference between cav-1 gene expression in the aggressive (PC3 and DU145) and non-aggressive (LNCaP and MDA PCa 2b) cell lines was statistically significant (p<0.05).

### Analysis of intracellular BODIPY-cholesterol efflux

**Figure 3.**
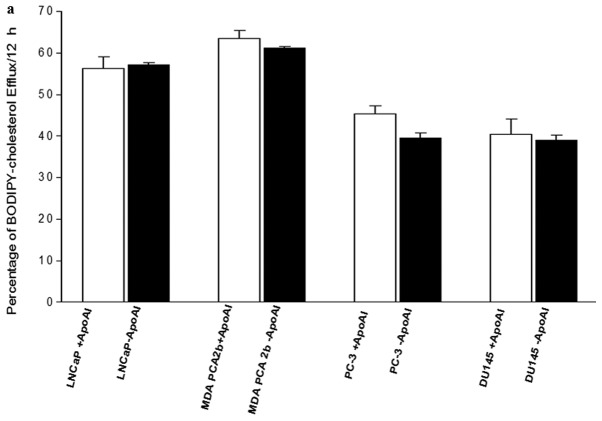

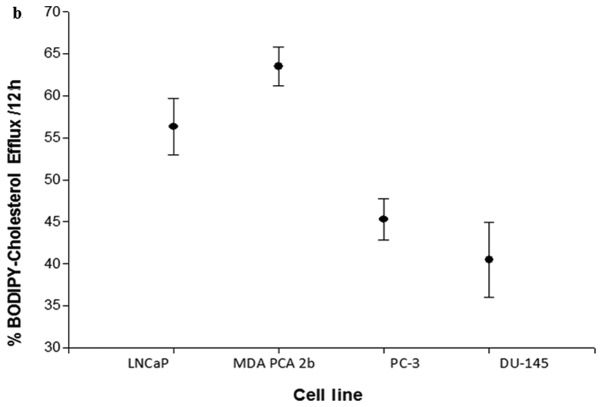

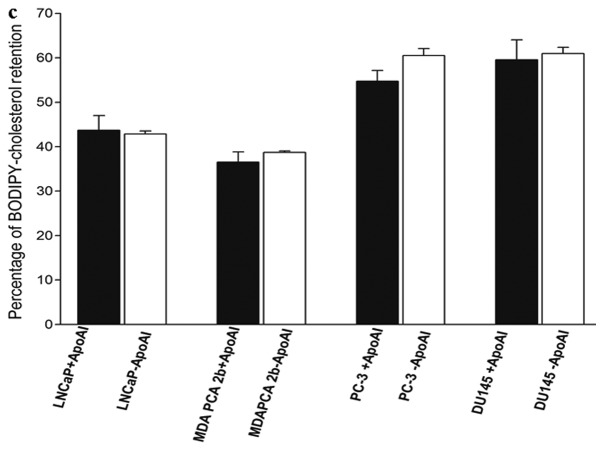
(a) Bar graph showing the percentage of BODIPY-cholesterol efflux from prostate cancer cell lines incubated with and without cholesterol acceptors (ApoA-I). The percentage of BODIPY-cholesterol efflux in cultures supplemented with and without cholesterol acceptors is significant (p<0.05). Also, the difference in BODIPY-cholesterol efflux between the non-aggressive and aggressive cell lines is highly significant (p<0.001). (b) Interval plots showing the percentage of BODIPY-cholesterol efflux from aggressive (PC-3 and DU145) and non-aggressive (LNCaP and MDA PCA 2b) prostate cancer cell lines. Plots determined from individual 95% CI for means based on pooled standard deviations. Differences in BODIPY-cholesterol efflux were determined by Tukey’s test. BODIPY-cholesterol efflux in aggressive cell lines (PC-3 and DU145) compared with non-aggressive cell lines (LNCaP and MDA PCA 2b): p<0.001; there was no difference in efflux among aggressive cell lines, and among non-aggressive cell lines. (c) Bar graph showing the percentage of BODIPY-cholesterol retention in cultures of prostate cancer cell lines incubated with and without cholesterol acceptors (ApoAI). The percentage of BODIPY-cholesterol retained in the presence and absence of cholesterol acceptors is significant (p<0.05). The mean percentage of BODIPY-cholesterol retained by non-aggressive and aggressive cell lines is highly significant (p<0.001).

Examination of the relationship between ApoE phenotypes and its cholesterol regulatory mechanism revealed a significantly (p<0.001) greater efflux of BODIPY-cholesterol from the non-aggressive cell lines compared to the aggressive cell lines ( Fig. 3a ). The statistical summary of the reverse cholesterol transport process showed a significant difference (p<0.001) in cholesterol efflux across all cell lines. Specifically, interval plots illustrating the differences in cholesterol efflux between aggressive and non-aggressive prostate cancer cell lines assessed with Tukey’s test ( Fig. 3b ) revealed a significantly higher cholesterol efflux in the non-aggressive prostate cancer cell lines (LNCap, 56.32±3.33% and MDA PCa 2b, 63.49±2.28%) than from the aggressive cell lines (PC-3, 45.29±2.40% and DU-145, 40.46±4.49%). It has been established that cells incubated in the presence of cholesterol acceptors such as ApoA-I release more cholesterol to the medium than those incubated without the acceptors. This is consistent with our data showing a statistically significant (p<0.05) difference between the mean values of cholesterol efflux from cells cultured in the presence and absence of cholesterol acceptors. There was no interaction (p>0.17) between prostate cancer cell lines and the presence or absence of cholesterol acceptors.

### Analysis of retained BODIPY-cholesterol

Consistent with the expected relationship between apoE phenotypes and intra-cellular cholesterol regulation, we observed a significantly higher (p<0.001) retention of cholesterol by the aggressive cell lines, compared to the non-aggressive cell lines ( Fig. 3c ) Likewise, the difference between cholesterol retention in the presence and absence of efflux acceptors in some of the cell lines was statistically significant (p<0.001).

### Microscopic analysis of membrane localized BODIPY-cholesterol

**Figure 4.**
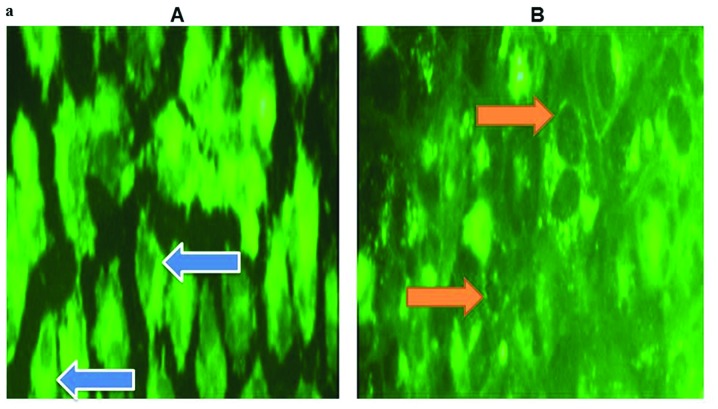

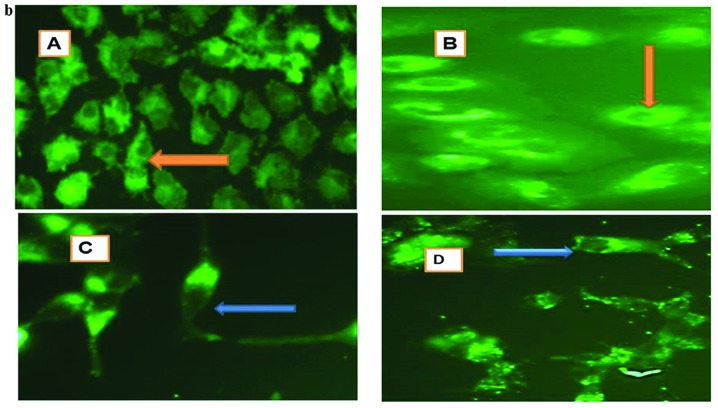
(a) Fluorescence microscopy showing differences in BODIPY-cholesterol localization and retention in a representative prostate cancer cell line (DU-145) cultured in the presence and absence of cholesterol acceptors. BODIPY-cholesterol-labeled cells incubated in: (A) the absence of cholesterol acceptor (left panel) and (B) the presence of cholesterol acceptor ApoA-I (right panel). Note the comparatively thinner membrane localized BODIPY-cholesterol (orange arrow) in cells treated with cholesterol acceptors (ApoA-I) in contrast to thicker membrane localized BODIPY-cholesterol (blue arrow) in the absence of cholesterol acceptors. (b) Fluorescence microscopy images showing a higher localization of BODIPY-cholesterol in membranes of aggressive prostate cancer cell lines compared to the non-aggressive cell lines. Plasma membranes of aggressive prostate cancer cell lines, DU145 (A) and PC3 (B), show higher intensity of membrane-localized BODIPY-cholesterol (orange arrows) compared to membrane-localized BODIPY-cholesterol (blue arrows) in non-aggressive prostate cancer cell lines, LNCaP (C) and MDA PCa 2b (D).

To probe the actual localization of the retained BODIPY-cholesterol using fluorescence microscopy, we observed higher membrane localization in of BODIPY-cholesterol in cell cultures lacking cholesterol acceptors ( Fig. 4a ). Finally, the fluorescence microscopy images showed a higher localization of BODIPY-cholesterol in membranes of aggressive prostate cancer cell lines, than those of non-aggressive ones ( Fig. 4b ).

## Discussion

In this study, we explored the association between ApoE phenotypes of prostate cancer cell lines and the risk of aggressive prostate cancer. We investigated PC3 and DU145, the two established androgen-insensitive cell lines that are highly invasive and tumorigenic in athymic nude mice, and uniquely express PROS1, a crucial protein mediator of cancer progression, thus justifying their consistently undisputed characterization as hallmarks of aggressiveness ( 33 , 34 ) . In contrast, LNCaP and MDA PCa 2b, which are androgen receptor-expressing, androgen-refractory, poorly migratory and less invasive cell lines that universally characterize non-aggressiveness were also investigated ( 33 , 35 – 39 ) . Accordingly, we classified tumor aggressiveness by regarding the highly tumorigenic and the moderately tumorigenic prostate cancer cell lines (PC3 and DU145, respectively) as ‘aggressive’; while the weakly tumorigenic cell lines (LNCaP and MDAPCa 2b) ( 33 ) were regarded as ‘non-aggressive’. We observed that the aggressive cell lines were heterozygous for the Apoε2/ε4 genotype, while the non-aggressive cell lines were heterozygous carriers of at least one Apoε3 genotype. The complementary alleles of the non-aggressive cell lines were Apoε3 (PC3) and Apoε4 (MDAPCa 2b). Thus, PC3 was homozygous for the ApoE3/E3 phenotype, while MDAPCa 2b was heterozygous for the ApoE3/E4 phenotype.

To the best of our knowledge, the genetic polymorphism of ApoE in prostate cancer cell lines has not been previously documented. Rather, an earlier investigation focused on the linear relationship between the expression of ApoE mRNA and the aggressiveness of prostate cancer cell lines ( 40 ) . Remarkably, a number of previous investigations had investigated the relationship between genetic polymorphism of ApoE and susceptibility to breast ( 41 ) and prostate tumors ( 42 ) . While these studies were generally focused on the transcriptional regulation of tumor aggressiveness by ApoE, the present study exclusively investigated the relationship between ApoE variants and acknowledged aggressive prostate cancer cell lines. Accordingly, we observed that the aggressive prostate cancer cell lines carried the ApoE2/E4 phenotype, while the non-aggressive types carried the ApoE3/E3 or E3/ E4 phenotypes. Although there is scarcely any data on the allelic variation of prostate cancer cell lines, an earlier study in human subjects revealed overexpressed Apoε2/ε4 alleles in hormone-refractory, locally recurrent prostate cancer patients as compared to control subjects ( 43 ) . The observed carriage of the Apoε2/ε4 alleles in these patients and our demonstration of its frequency in aggressive prostate cancer cell lines highlight its probable role in aggressive disease, since hormone-refractory and recurrent prostate carcinomas are regarded as clinically aggressive ( 44 ) .

Currently, there are no clearly defined mechanisms to explain the relationship between the ApoE isoforms, especially the ApoE2/E4 phenotype and aggressive prostate cancer. However, a possible link could be inferred from our observed accumulation of intracellular cholesterol in cells carrying certain ApoE phenotypes. A preponderance of evidence suggests that intracellular cholesterol overload supports the progression of prostate cancer to advanced disease ( 45 ) . In this context, the role of ApoE in clearing circulating cholesterol is the regulation of its influx to cells ( 18 ) , and its efflux by reverse cholesterol transport, involving its extraction from peripheral tissues to the liver for excretion ( 46 ) . Phenotypic differences in the regulation of cholesterol efflux from cells by ApoE ( 46 , 47 ) , may govern cholesterol accumulation in macrophages and RAW 264.7 cell lines ( 47 , 48 ) . Although we are not aware of such a relationship in prostate cancer cell lines, the concept is consistent with our findings that the three ApoE phenotypes in our prostate cancer cell lines have distinct abilities to promote cholesterol efflux, and to inversely accumulate cholesterol.

The Apoε3 allele-carrying non-aggressive cell lines, or precisely the LNCaP which carries the ApoE3/E3 phenotype, and the MDA PCa 2b, which carries the ApoE3/E4 phenotype displayed higher BODIPY-cholesterol efflux, while the aggressive cell lines carrying the ApoE2/E4 phenotype (PC3 and DU145) displayed lower BODIPY-cholesterol efflux. There was no significant difference in the BODIPY-cholesterol efflux of the poorly aggressive cell lines, and neither was there any difference in that of the aggressive cell lines. To support the isoform-mediated differences in cellular cholesterollevels, an assessment of BODIPY-cholesterol accumulation by fluorescence microscopy revealed that the non-aggressive prostate cancer cell lines, which carry at least one Apoε3 allele retained less membrane cholesterol, in contrast to the Apoε3 allele-deficient aggressive cell lines. These data appear consistent with the high expression of key effectors of cholesterol production and downregulation of the expression of major cholesterol exporters in prostate cancer cells, thus supporting the reprogramming of their cholesterol metabolism to favor its increased production and rapid cell growth ( 48 ) . Our data revealed a higher BODIPY-cholesterol within membranes of the aggressive prostate cancer cell lines, which carry the ApoE2/E4 phenotype, while a lower amount was found in membranes of the non-aggressive prostate cancer cell lines, which carry at least one Apoε3 allele.

Taken together, these exploratory findings support the premise that in prostate cancer cell lines, efficient cholesterol efflux and its resultant depletion is associated with certain ApoE isoforms. To the contrary, our data imply that other ApoE isoforms are associated with deregulated cholesterol efflux, and membrane accumulation. Consistent with this, our results suggest that aggressive prostate cancer cell lines, carrying the ApoE2/E4 phenotype, exhibited low BODIPY-cholesterol efflux and accumulated BODIPY-cholesterol in the membranes.

To our knowledge there is paucity of data on how the different ApoE isoforms influence cholesterol efflux in prostate cancer cell lines. Nevertheless, studies from human intact fibroblasts showed that lipoprotein-containing ApoE particles (γ-LpE) from ApoE3/E3 individuals stimulated 7- to 13-fold more cholesterol efflux than from ApoE2/E2 or ApoE4/E4 individuals ( 46 ) . Earlier studies using RAW 264.7 mouse macrophage cells did not find any difference in cholesterol efflux from cells with the three ApoE isoforms ( 49 ) . However, all the clonal macrophage cell lines used in that study carried three different homozygous ApoE-isoforms, whereas our investigated prostate cancer cell lines had a combination of heterozygous and homozygous ApoE isoforms. Again, the differences in the ApoE isoform-dependent cholesterol efflux in RAW 264.7 cells may have been annulled by the presence in the culture of a cAMP analogue, which induces a cellular ApoE receptor-mediated transfer of cholesterol to all apolipoproteins, followed by the subsequent release of the lipoprotein particles ( 49 ) .

ApoE influences cholesterol efflux from cells by reverse cholesterol transport, and rids cells of excess cholesterol, which is transported to the liver for excretion. To influence this cholesterol efflux, ApoE is generally synthesized in various cell types including the prostate, and then transported to the plasma membrane for secretion ( 23 ) . In cholesterol-rich cells, this secreted ApoE promotes cholesterol efflux even in the absence of cholesterol acceptors ( 23 ) . The exact mechanism by which ApoE mediates cellular cholesterol efflux and whether this cholesterol efflux is isoform-specific is still unknown. The most consistent explanation for ApoE-mediated cholesterol efflux had relied on the intracellular association of ApoE with cAMP-induced ABCA1, which induces increased secretion of ApoE and catalysis of an initial transfer of cholesterol to the lipid poor ApoE ( 16 ) . Doubt has been cast on the sole regulation of cholesterol efflux by ABCA1, leading to the recognition of ABCG1 as finalizing the full transfer of this cholesterol to the apolipoprotein ( 16 ) . One reason cited for the plausibility of the ApoE isoform-dependent cholesterol efflux was the low affinity of the apoE2 isoform versus the ApoE3 or ApoE4 isoforms for heparan sulfate proteoglycans (HSPGs), which modulate cholesterol depleting ability of ApoE ( 50 ) . The higher cholesterol efflux of ApoE2-carrying cells was explained by several demonstrations that the higher affinity of ApoE3 and ApoE4 for HSPGs caused the sequestration of the latter isoforms into the pericellular proteoglycan matrix, leading to their eventual cellular degradation ( 47 , 50 ) . Thus, ApoE2 is the most effective for cholesterol efflux, followed by ApoE3, while ApoE4 is least effective. The effective cholesterol efflux of the ApoE2/E2 phenotype-carrying cells is consistent with an earlier observation that cholesterol loaded cells redirect this ApoE isoform from the degradatory to the secretory pathway ( 47 ) . This study also revealed that cells carrying the ApoE3/E3 phenotype secreted nearly 77% of ApoE proteins, leading to reduced intracellular cholesterol accumulation ( 47 ) . These investigations concluded that ApoE4/ E4-carrying cells secreted the most ApoE apolipoproteins, but lacked effective net cholesterol efflux because of greater HSPGs affinity and re-uptake of cholesterol particles ( 47 , 50 ) .

Our observed reduction in cholesterol efflux and increased cholesterol retention by the ApoE2/E4 phenotype-carrying prostate cancer cell lines highlights the dominance of the ε4 allele over the ε2 allele in heterozygosity. Elucidating how the ε4 allele dominates the ε2 allele in cholesterol transport will significantly improve our understanding of the isoform-dependent regulation of cholesterol efflux and retention. The preceding discussion is consistent with previous evidence showing that reduced cholesterol efflux by ApoE contributes to membrane cholesterol accumulation ( 51 ) . The current paradigm in cholesterol homeostasis holds that the increased prevalence of lipid rafts and caveolae in cells is attributable to membrane cholesterol accumulation. Cav-1 is the most celebrated structural protein of the caveolae that binds cholesterol, and its absence impairs cholesterol homeostasis ( 52 ) . Additionally, membrane cholesterol enrichment has been associated with increased cav-1 mRNA expression ( 53 ) . This is consistent with our studies showing that BODIPY-cholesterol-accumulating and aggressive prostate cancer cell lines overexpress cav-1 mRNA, whereas BODIPY-cholesterol poor and non-aggressive prostate cancer cell lines express low or no cav-1 mRNA.

It has been previously demonstrated that cav-1 expression is highly dependent on the availability of cholesterol ( 54 ) , and its depletion diminishes the caveolae by removing cav-1 from the membrane ( 55 ) . We observed a higher retention of the fluorescent cholesterol analogue in the aggressive prostate cancer cell lines, which concurrently overexpressed cav-1 mRNA, further strengthening the relationship between cav-1 expression and cell aggression. This relationship supports earlier demon stration that cav-1 is a potential biomarker of aggressive prostate cancer ( 56 ) . To the contrary, we found no expression of cav-1 mRNA in the non-aggressive prostate cancer cell lines. This finding is consistent with previous studies indicating the overexpression of cav-1 in mouse and human metastatic prostate cancer cells ( 57 ) . This has been corroborated by a recent report indicating that the absence of cav-1 significantly inhibited the progression of prostate cancer to highly invasive and metastatic disease ( 58 ) . Overall, molecular approaches highlight the plausible relationship between intracellular cholesterol accumulation and prostate cancer. Pathological studies of prostate tumor samples had provided evidence for such relationship by demonstrating a positive correlation between Gleason grade and the levels of expression of this cholesterol-binding protein or cav-1 ( 55 ) .

Although the role of caveolae in the progression of solid tumors is not fully understood, the contribution of cav-1 to signaling processes that initiate prostate cancer has been extensively investigated. The initiation and progression of cancer by cav-1 is linked to its tendency to form platforms that aggregate membrane proteins for cell proliferation signaling. This function of cav-1 is partly related to its possession of an amino acid domain that binds a variety of signaling proteins ( 49 ) , which recently includes ABCA1 and ApoE ( 32 , 59 ) . Binding of these proteins to cav-1 is believed to inactivate downstream growth signals ( 59 ) . An interaction between cav-1, ABCA1 and ApoE is appropriate as it suggests crosstalk among them favoring cholesterol balance, reduced cholesterol overload and inhibition of cell proliferation. However, the effect of cav-1 expression on cholesterol efflux is still controversial and has been attributed to differences in the cell types used for such studies ( 60 ) . The molecular mechanism by which these membrane-anchored proteins maintain the correct intracellular cholesterol balance and the particular ApoE isoforms that perturb such equilibrium has not been elucidated. Our results highlight the need for further experiments to confirm whether the ApoE2/E4 is the dysfunctional phenotype that inhibits cholesterol efflux, leading to prostate cancer cell proliferation.

In summary, our data suggest the possibility of the relationship between the ApoE2/E4 phenotype, the intracellular cholesterol efflux and cholesterol content of prostate cancer cells. This is consistent with our observed overexpression of cav-1, the sentinel gene for cholesterol overload exclusively by the ApoE2/E4-carrying aggressive prostate cancer cell lines.

We conclude that the overexpression of cav-1 and cholesterol overload in aggressive prostate cancer cell lines, suggests that cav-1 may confer survival advantage on prostate cancer cells leading to disease progression. The less than anticipated aggressiveness of the African American cancer cell line (MDA PCa 2b) suggests that the Apoε3 allele masks the deregulated cholesterol balance associated with the ancestral Apoε4 allele. The low cholesterol efflux and higher cholesterol retention in ApoE2/E4 phenotype-carrying aggressive prostate cancer cells justifies further investigation of ApoE2/E4 phenotype as a biomarker of aggressive disease. While the evidence amassed so far and elsewhere clearly indicates that genetic factors play a key role in determining the susceptibility to aggressive prostate cancer, the sample size in our study imposes limited statistical power and restrains definitive conclusions. Unraveling the mechanism by which the dysfunctional apoE isoforms transforms the prostate cancer cell lines to aggressive phenotypes could be a daunting task, which however could be overcome by genetic manipulation under varying physiological conditions, and may provide new insights into the pathogenesis and therapeutic targets of the disease.

## References

[1] 
Williams
 
SD
, 
Cook
 
ED
, 
Anderson
 
KB
, 
Hamilton
 
SJ
 (
2008
). Bridging the gap between populations: the challenge of reducing cancer disparities among African-American and other ethnic minority populations. US Oncol.

[2] 
Ifere
 
GO
, 
Abebe
 
F
, 
Ananaba
 
GA
 (
2012
). Emergent trends in the reported incidence of prostate cancer in Nigeria. Clin Epidemiol.

[3] 
Sakr
 
WA
 (
1999
). Prostatic intraepithelial neoplasia: A marker for high-risk groups and a potential target for chemoprevention. Eur Urol.

[4] 
Bock
 
CH
, 
Powell
 
I
, 
Kittles
 
RA
, 
Hsing
 
AW
, 
Carpten
 
J
 (
2011
). Racial disparity in prostate cancer incidence, biochemical recurrence, and mortality. Prostate Cancer.

[5] 
Bigler
 
SA
, 
Pound
 
CR
, 
Zhou
 
X
 (
2011
). A retrospective study on pathological features and racial disparities in prostate cancer. Prostate Cancer.

[6] 
Lee
 
J
, 
Demissie
 
K
, 
Lu
 
SE
, 
Rhoads
 
GG
 (
2007
). Cancer incidence among Korean-American immigrants in the United States and native Koreans of South Korea. Cancer Control.

[7] 
Ma
 
RW
, 
Chapman
 
K
 (
2009
). A systematic review of the effect of diet in prostate cancer prevention and treatment. J Hum Nutr Diet.

[8] 
Michaud
 
DS
, 
Augustsson
 
K
, 
Rimm
 
EB
, 
Stampfer
 
MJ
, 
Willet
 
WC
, 
Giovanucci
 
E
 (
2001
). A prospective study on intake of animal products and risk of prostate cancer. Cancer Causes Control.

[9] 
Waddell
 
WJ
 (
1998
). Epidemiological studies and effects of environmental estrogens. Int J Toxicol.

[10] 
Le Marchand
 
L
, 
Wilkens
 
LR
 (
2008
). Design considerations for genomic association studies: importance of gene-environment interactions. Cancer Epidemiol Biomarkers Prev.

[11] 
Nothlings
 
U
, 
Yamamoto
 
JF
, 
Wilkens
 
LR
, 
Murphy
 
SP
, 
Park
 
SY
, 
Henderson
 
BE
, 
Kolonel
 
LN
, 
Le Marchand
 
L
 (
2009
). Meat and heterocyclic amine intake, smoking, NAT1 and NAT2 polymorphisms, and colorectal cancer risk in the multiethnic cohort study. Cancer Epidemiol Biomarkers Prev.

[12] 
Wünsch Filho
 
V
, 
Zago
 
MA
 (
2005
). Modern cancer epidemiology research: genetic polymorphism and environment. Rev Saude Publica.

[13] 
Finch
 
CE
, 
Stanford
 
CB
 (
2004
). Meat-adaptive genes and the evolution of slower aging in humans. Q Rev Biol.

[14] 
Mahley
 
RW
 (
1988
). Apolipoprotein E: cholesterol transport protein with expanding role in cell biology. Science.

[15] 
Tziakas
 
DN
, 
Chalikias
 
GK
, 
Antonoglou
 
CO
, 
Veletza
 
S
, 
Tentes
 
IK
, 
Kortsaris
 
AX
, 
Hatseras
 
DI
, 
Kaski
 
JC
 (
2006
). Apolipoprotein E genotype and circulating interleukin-10 levels in patients with stable and unstable coronary artery disease. J Am Coll Cardiol.

[16] 
Leduc
 
V
, 
Domenger
 
D
, 
De Beaumont
 
L
, 
Lalonde
 
D
, 
Bélanger-Jasmin
 
S
, 
Poirier
 
J
 (
2011
). Function and comorbidities of apolipoprotein E in Alzheimer’s disease. Int J Alzheimers Dis.

[17] 
Eichner
 
JE
, 
Dunn
 
ST
, 
Perveen
 
G
, 
Thompson
 
DM
, 
Stewart
 
KE
, 
Stroehla
 
BC
 (
2002
). Apolipoprotein E polymorphism and cardiovascular disease: a HuGE review. Am J Epidemiol.

[18] 
Mahley
 
RW
, 
Rall
 
SC
 (
2000
). Apolipoprotein E: far more than a lipid transport protein. Annu Rev Genomics Hum Genet.

[19] 
Moore
 
RJ
, 
Chamberlain
 
RM
, 
Khuri
 
FR
 (
2004
). Apolipoprotein E and the risk of breast cancer in African-American and non-Hispanic white women. A review. Oncology.

[20] 
Kallio
 
MJ
, 
Salmenperä
 
L
, 
Siimes
 
MA
, 
Perheentupa
 
J
, 
Gylling
 
H
, 
Miettinen
 
TA
 (
1997
). Apolipoprotein E phenotype determines serum cholesterol in infants during both high-cholesterol breast feeding and low-cholesterol formula feeding. J Lipid Res.

[21] 
Berglund
 
L
 (
2001
). The APOE gene and diets - food (and drink) for thought. Am J Clin Nutr.

[22] 
Papaioannou
 
I
, 
Simmons
 
JP
, 
Owen
 
JS
 (
2012
). Targeted in situ gene correction of dysfunctional APOE alleles to produce atheroprotective plasma ApoE3 protein. Cardiol Res Pract.

[23] 
Kockx
 
M
, 
Jessup
 
W
, 
Kritharides
 
L
 (
2008
). Regulation of endogenous apolipoprotein E secretion by macrophages. Arterioscler Thromb Vasc Biol.

[24] 
Huang
 
ZH
, 
Fitzgerald
 
ML
, 
Mazzone
 
T
 (
2006
). Distinct cellular loci for the ABCA1-dependent and ABCA1-independent lipid efflux mediated by endogenous apolipoprotein E expression. Arterioscler Thromb Vasc Biol.

[25] 
Huang
 
Y
, 
von Eckardstein
 
A
, 
Wu
 
S
, 
Assmann
 
G
 (
1995
). Effects of the apolipoprotein E polymorphism on uptake and transfer of cell derived cholesterol in plasma. J Clin Invest.

[26] 
Lane
 
RM
, 
Farlow
 
MR
 (
2005
). Lipid homeostasis and apolipoprotein E in the development and progression of Alzheimer’s disease. J Lipid Res.

[27] 
Uittenbogaard
 
A
, 
Ying
 
Y
, 
Smart
 
EJ
 (
1998
). Characterization of a cytosolic heat-shock protein-caveolin chaperone complex. Involvement in cholesterol trafficking. J Biol Chem.

[28] 
Liscum
 
L
, 
Munn
 
NJ
 (
1999
). Intracellular cholesterol transport. Biochim Biophys Acta.

[29] 
Li
 
YC
, 
Park
 
MJ
, 
Ye
 
SK
, 
Kim
 
CW
, 
Kim
 
YN
 (
2006
). Elevated levels of cholesterol-rich lipid rafts in cancer cells are correlated with apoptosis sensitivity induced by cholesterol-depleting agents. Am J Pathol.

[30] 
Hixson
 
JE
, 
Vernier
 
DT
 (
1990
). Restriction isotyping of human apolipoprotein E by gene amplification and cleavage with 
*
Hha
*
I. J Lipid Res.

[31] 
Sankaranarayanan
 
S
, 
Kellner-Weibel
 
G
, 
de la Llera-Moya
 
M
, 
Phillips
 
MC
, 
Asztalos
 
BF
, 
Bittmam
 
R
, 
Rothblat
 
GH
 (
2011
). A sensitive assay for ABCA1-mediated cholesterol efflux using BODIPY-cholesterol. J Lipid Res.

[32] 
Yue
 
L
, 
Mazzone
 
T
 (
2011
). Endogenous adipocyte apolipoprotein E is colocalized with caveolin at the adipocyte plasma membrane. J Lipid Res.

[33] 
Nair
 
HK
, 
Rao
 
KV
, 
Aalinkeel
 
R
, 
Mahajan
 
S
, 
Chawda
 
R
, 
Schwartz
 
SA
 (
2004
). Inhibition of prostate cancer cell colony formation by the flavonoid quercetin correlates with modulation of specific regulatory genes. Clin Diagn Lab Immunol.

[34] 
Saraon
 
P
, 
Musrap
 
N
, 
Cretu
 
D
, 
Karagiannis
 
GS
, 
Batruch
 
I
, 
Smith
 
C
, 
Drabovich
 
AP
, 
Trudel
 
D
, 
van der Kwast
 
T
, 
Morrissey
 
C
, 
Jarvi
 
KA
, 
Diamandis
 
EP
 (
2012
). Proteomic profiling of androgen-independent prostate cancer cell lines reveals a role for protein S during the development of high grade and castration-resistant prostate cancer. J Biol Chem.

[35] 
Wang
 
B
, 
Yang
 
Q
, 
Ceniccola
 
K
, 
Bianco
 
F
, 
Andrawis
 
R
, 
Jarret
 
T
, 
Frazier
 
H
, 
Patierno
 
SR
, 
Lee
 
NH
 (
2013
). Androgen receptor-target gene in African American prostate cancer disparities. Prostate Cancer.

[36] 
Kim
 
DH
, 
Wirtz
 
D
 (
2011
). Recapitulating cancer cell invasion in vitro. Proc Natl Acad Sci USA.

[37] 
Hoosein
 
NM
 (
1998
). Neuroendocrine and immune mediators in prostate cancer progression. Front Biosci.

[38] 
Hara
 
T
, 
Miyazaki
 
H
, 
Lee
 
A
, 
Tran
 
CP
, 
Reiter
 
RE
 (
2008
). Androgen receptor and invasion in prostate cancer. Cancer Res.

[39] 
Nomura
 
DK
, 
Lombardi
 
DP
, 
Chang
 
JW
, 
Niessen
 
S
, 
Ward
 
AM
, 
Long
 
JZ
, 
Hoover
 
HH
, 
Cravatt
 
BF
 (
2011
). Monoacylglycerol lipase exerts dual control over endocannabinoid and fatty acid pathways to support prostate cancer. Chem Biol.

[40] 
Venanzoni
 
MC
, 
Giunta
 
S
, 
Murara
 
GB
, 
Storari
 
L
, 
Crescini
 
C
, 
Mazzucchelli
 
R
, 
Montironi
 
R
, 
Seth
 
A
 (
2003
). Apolipoprotein E expression in localized prostate cancers. Int J Oncol.

[41] 
Saadat
 
M
 (
2012
). Apolipoprotein E (ApoE) polymorphism and susceptibility to breast cancer: a meta-analysis. Cancer Res Treat.

[42] 
Niemi
 
M
, 
Kervinen
 
K
, 
Kiviniemi
 
H
, 
Lukkarinen
 
O
, 
Kyllönen
 
AP
, 
Apaja-Sarkkinen
 
M
, 
Savolainen
 
MJ
, 
Kairaluoma
 
MI
, 
Kesäniemi
 
YA
 (
2000
). Apolipoprotein E phenotype, cholesterol and breast and prostate cancer. J Epidemiol Community Health.

[43] 
Haapla
 
K
, 
Lehtimäki
 
T
, 
Ilveskoski
 
E
, 
Koivisto
 
PA
 (
2000
). Apolipoprotein E is not linked to locally recurrent hormone-refractory prostate cancer. Prostate Cancer Prostatic Dis.

[44] 
Koivisto
 
P
, 
Visakorpi
 
T
, 
Rantala
 
I
, 
Isola
 
J
 (
1997
). Increased cell proliferation activity and decreased cell death are associated with the emergence of hormone-refractory recurrent prostate cancer. J Pathol.

[45] 
Mostaghel
 
EA
, 
Solomon
 
KR
, 
Pelton
 
K
, 
Freeman
 
MR
, 
Montgomery
 
RB
 (2012). Impact of circulating cholesterol levels on growth and intratumoral androgen concentration of prostate tumors. PLoS One.

[46] 
Krimbou
 
L
, 
Denis
 
M
, 
Haidar
 
B
, 
Carrier
 
M
, 
Marci
 
M
, 
Genest
 
J
 (
2004
). Molecular interactions between apoE and ABCA1: impact on apoE lipidation. J Lipid Res.

[47] 
Cullen
 
P
, 
Cignarella
 
A
, 
Brennhausen
 
B
, 
Mohr
 
S
, 
Assmann
 
G
, 
Von Eckardstein
 
A
 (
1998
). Phenotype-dependent differences in apolipoprotein E metabolism and in cholesterol homeostasis in human monocyte-derived macrophages. J Clin Invest.

[48] 
Murtola
 
TJ
, 
Syvälä
 
H
, 
Pennanen
 
P
, 
Bläuer
 
M
, 
Solakivi
 
T
, 
Ylikomi
 
T
, 
Tammela
 
TL
 (2012). The importance of LDL and cholesterol metabolism for prostate epithelial cell growth. PLoS One.

[49] 
Smith
 
JD
, 
Miyata
 
M
, 
Ginsberg
 
M
, 
Grigaux
 
C
, 
Shmookler
 
E
, 
Plump
 
AS
 (
1996
). Cyclic AMP induces apolipoprotein E binding activity and promotes cholesterol efflux from a macrophage cell line to apolipoprotein acceptors. J Biol Chem.

[50] 
Hara
 
M
, 
Matsushima
 
T
, 
Satoh
 
H
, 
Iso-o
 
N
, 
Noto
 
H
, 
Togo
 
M
, 
Kimura
 
S
, 
Hashimoto
 
Y
, 
Tsukamoto
 
K
 (
2003
). Isoform-dependent cholesterol efflux from macrophages by apolipoprotein E is modulated by cell surface proteoglycan. Arterioscler Thromb Vasc Biol.

[51] 
Karasinska
 
JM
, 
Hayden
 
MR
 (
2011
). Cholesterol metabolism in Huntington disease. Nat Rev Neurol.

[52] 
Fernández-Hernando
 
C
, 
Yu
 
J
, 
Dávalos
 
A
, 
Prendergast
 
J
, 
Sessa
 
WC
 (
2010
). Endothelial-specific overexpression of caveolin-1 accelerates atherosclerosis in apolipoprotein E-deficient mice. Am J Pathol.

[53] 
Cohen
 
AW
, 
Hnaska
 
R
, 
Schubert
 
W
, 
Lisanti
 
MP
 (
2004
). Role of caveolae and caveolins in health and disease. Physiol Rev.

[54] 
Frank
 
PG
, 
Cheung
 
MW
, 
Pavlides
 
S
, 
Llaverias
 
G
, 
Park
 
DS
, 
Lisanti
 
MP
 (
2006
). Caveolin-1 and regulation of cellular cholesterol homeostasis. Am J Physiol Heart Circ Physiol.

[55] 
Daniel
 
EE
, 
El-Yazbi
 
A
, 
Cho
 
WJ
 (
2006
). Caveolae and calcium handling, a review and a hypothesis. J Cell Mol Med.

[56] 
Yang
 
G
, 
Truong
 
LD
, 
Wheeler
 
TM
, 
Thompson
 
TC
 (
1999
). Caveolin-1 expression in clinically confined human prostate cancer: a novel prognostic marker. Cancer Res.

[57] 
Thompson
 
TC
 (
1999
). Metastasis-related genes in prostate cancer: the role of caveolin-1. Cancer Metastasis Rev.

[58] 
Williams
 
TM
, 
Hassan
 
GS
, 
Li
 
J
, 
Cohen
 
AW
, 
Medina
 
F
, 
Frank
 
PG
, 
Pestell
 
RG
, 
Di Vizio
 
D
, 
Loda
 
M
, 
Lisanti
 
MP
 (
2005
). Caveolin-1 promotes tumor progression in an autochthonous mouse model of prostate cancer. J Biol Chem.

[59] 
Lin
 
YC
, 
Ma
 
C
, 
Hsu
 
WC
, 
Lo
 
HF
, 
Yang
 
VC
 (
2007
). Molecular interaction between caveolin-1 and ABCA1 on high-density lipoprotein-mediated cholesterol efflux in aortic endothelial cells. Cardiovasc Res.

[60] 
Le Lay
 
S
, 
Rodriguez
 
M
, 
Jessup
 
W
, 
Rentero
 
C
, 
Li
 
Q
, 
Cartland
 
S
, 
Grewal
 
T
, 
Gaus
 
K
 (2011). Caveolin-1-mediated apolipoprotein A-I membrane binding sites are not required for cholesterol efflux. PLoS One.

